# AFAP1 antisense RNA 1 promotes retinoblastoma progression by sponging microRNA miR-545-3p that targets G protein subunit beta 1

**DOI:** 10.1080/21655979.2022.2033464

**Published:** 2022-02-22

**Authors:** Wenting Tang, Li Zhang, Jing Li, Yu Guan

**Affiliations:** a Department of Ophthalmology, The First Affiliated Hospital of Chengdu Medical College, Chengdu, China; b Department of Ophthalmology, Nuclear Industry 416th Hospital, Chengdu, China

**Keywords:** ceRNA, retinoblastoma, lncRNA, cellule phenotypes

## Abstract

The oncogenic role of actin filament-associated protein 1 antisense RNA 1 (AFAP1-AS1) has been reported in retinoblastoma (RB). However, the underlying regulatory mechanisms remain poorly understood. In this study, reverse transcription-quantitative polymerase chain reaction (RT-qPCR) and Western blotting were performed to analyze the expression of AFAP1-AS1, microRNA miR-545-3p, or G protein subunit beta 1 (GNB1). Cell Counting Kit-8 (CCK-8) and Transwell migration assays were used to detect cell proliferation and migration. In addition, caspase-3 activity was monitored by caspase-3 activity assay. Luciferase reporter assays combined with RNA immunoprecipitation (RIP) and pull-down assays were performed to elucidate the target relationship between miR-545-3p and AFAP1-AS1 or GNB1. Xenograft tumor experiments were performed to evaluate RB cell growth *in vivo*. Increased AFAP1-AS1 and GNB1 expression in RB tissues and cells was confirmed by RT-qPCR; conversely, miR-545-3p was found to be downregulated in RB tissues and cells. AFAP1-AS1 overexpression resulted in increased proliferation and migration of RB cells, whereas AFAP1-AS1 silencing resulted in decreased proliferation and migration of RB cells. Moreover, AFAP1-AS1 was found to target miR-545-3p. The anti-miR-545-3p treatment phenocopied the effect of AFAP1-AS1 overexpression and promoted RB cell growth *in vivo*. miR-545-3p was found to directly target GNB1. GNB1 silencing resulted in reduced proliferation and migration of RB cells and attenuated the oncogenic effect of the miR-545-3p inhibitor. Thus, in this study, a novel ceRNA regulation network of AFAP1-AS1 in RB was identified, where AFAP1-AS1 regulated GNB1 expression by targeting miR-545-3p, ultimately driving RB progression.

## Introduction

Retinoblastoma (RB) is an intraocular tumor originating from primitive stem cells within the retina and is characterized by leukorrhea and strabismus [1]. It is the most common intraocular malignant tumor in infants and young children, accounting for 3% of all malignant tumors occurring in infants and young children. In addition to surgical resection, systemic therapies combined with local ophthalmic artery intervention are highly recommended as treatment modalities for RB [2]. However, due to the age of onset being below 3 years, the diagnosis and treatment of RB are delayed, resulting in higher death rates. Current evidence has highlighted the role played by heredity and genetic alterations in RB pathogenesis [3]. Therefore, further exploration of the regulatory mechanism of RB is required to identify diagnostic and therapeutic targets.

Noncoding RNAs (ncRNAs) are a family of non-protein-coding transcripts. They are actively involved in the regulation of gene expression, multifaceted cell functions, such as cell growth and migration, and other cell phenotypes. Increasing evidence has shown that the deregulation of ncRNAs is tightly associated with the initiation and progression of cancers, including RB [4,5]. Micro RNAs (miRNAs) are a subset of ncRNAs, which have attracted much attention. miRNAs can post-transcriptionally suppress the expression of target tumor suppressor and promoter genes, and have therefore been implicated in cancer progression [6]. Emerging evidence has established a tight association between RB progression and long non-coding RNAs (lncRNAs; another type of ncRNA) [4]. lncRNAs construct an intricate network of interplays with various diverse biomolecules (DNA, RNA, or protein), and thus, their perturbation exhibits profound regulatory influences on RB pathologies. lncRNAs function as endogenous miRNA sponges to intervene in miRNA-mediated targeted mRNA expression, disrupting equilibrium in these regulatory mechanisms and the subsequent functional outcomes [7–9]. For example, the downregulation of plasmacytoma variant translocation 1 (PVT1) suppresses the malignant behavior of RB cells by enhancing the action of miR-488-3p [10]. Urothelial cancer-associated 1 (UCA1) sponges miR-513a-5p and increases RB cell proliferation and multidrug resistance [11]. AFAP1-AS1 is reportedly expressed in various cancers, including RB [12]. Zhong et al. and Sun et al. have demonstrated that AFAP1-AS1 modulates miR-545-3p with competing endogenous RNA (ceRNA) activity to drive cancer progression [13,14]. However, the synergistic interaction between AFAP1-AS1 and miR-545-3p in RB was underestimated.

In this study, following bioinformatic analysis, we found that G protein subunit beta 1 (GNB1) attracted our interest. GNB1 genes encode one of the subunits of heterotrimeric guanine nucleotide-binding proteins, which are responsible for integrating receptors and effector proteins and interfere with intracellular pathogen recognition. A few studies have revealed the effects of GNB1 in cancer. Chen et al. [15] found abnormal expression of GNB1 in clear cell renal cell carcinoma using the TCGA dataset, the Human Protein Atlas, and immunohistochemical staining and found that GNB1 expression was correlated with lymph node invasion, tumor grade, and tumor stage. Moreover, other studies have shown that GNB1 is overexpressed in cervical squamous cell carcinoma, which predicts poor prognosis in patients [16]. In addition, GNB1 is highly expressed in lung cancer and promotes the viability of lung cancer cells *in vitro* [17]. However, its role and regulatory mechanisms in RB have rarely been explored.

This study found the significance of AFAP1-AS1 in RB progression *in vitro*. Moreover, based on bioinformatics analyses and *in vitro* assays, we hypothesized that the AFAP1-AS1/miR-545-3p/GNB1 axis affects the uncontrolled proliferation and migration of RB cells during RB malignancy. This study aims to provide a valuable theoretical basis for the clinical diagnosis and treatment of RB.

## Materials and methods

### Bioinformatics analysis

GSE141208 was downloaded from GEO Datasets for screening the differentially expressed miRNAs in RB with P < 0.05, as well as the miRNAs binding to AFAP1-AS1 were predicted by starbase algorithm (https://starbase.sysu.edu.cn/index.php). Then, the target genes of miRNA were also predicted by starbase algorithm. Besides, GSE97508 was also downloaded from GEO Datasets for screening the differentially expressed genes (DEGs) with adjusted P (adj.P)<0.05 and |logFC|≥1.5. Finally, the protein-protein interaction network of screened DEGs was constructed by STRING (https://string-db.org/).

### Clinical sample

Samples of RB tissues and corresponding uncancerous eye tissues were intraoperatively collected from 24 patients diagnosed with RB at the Nuclear Industry 416^th^ Hospital, Chengdu 610,051, Sichuan, China between 2018 and 2019. Written informed consent was obtained from all the participants. Ethical approval was approved by the local Ethics Committee (Approval number: 2019CYFYHEC-BA-63).

### Cell culture and transfection

Human retinal pigment epithelial cells (ARPE-19) and three human RB cell lines (SO-Rb50, Y-79, and HXO-RB44) were obtained from ATCC, USA. ARPE-19, SO-Rb50, and HXO-RB44 cells were seeded in DMEM, and WeRI-Rb-1 and Y-79 in RPMI-1640 containing 10% fetal bovine serum (FBS) and 1% penicillin/streptomycin. All cells were maintained at 37°C with 5% CO_2_.

AFAP1-AS1 siRNA, GNB1 siRNA, and siRNA-NC were purchased from Hanheng Biotech Co. Ltd. (Shanghai, China) for AFAP1-AS1 silencing and were termed si-AFAP1-AS1, si-GNB1, and si-NC, respectively. The recombinant pcDNA3.1 constructs containing the full-length sequence were obtained from ThermoFisher (Waltham, MA, USA) for AFAP1-AS1 overexpression and was termed as OE-AFAP1-AS1. The empty pcDNA3.1 vectors was used as a negative control (OE-NC). Si-AFAP1-AS1, OE-AFAP1-AS1, si-GNB1, si-NC, or OE-NC were infected in 70% confluent Y-79 and WERI-Rb-1 cells for 48 h using a standard calcium phosphate transfection protocol. Thereafter, 20 μM puromycin was added to select the positive cell clones. After 5 days, the puromycin-resistant cells were collected and verified by reverse transcription-quantitative polymerase chain reaction (RT-qPCR) and Sanger sequencing. In addition, the miR-545-3p inhibitor and inhibitor NC, obtained from Shanghai GenePharma Co. Ltd. (Shanghai, China), were introduced into 80% confluent Y-79 and WERI-Rb-1 cells using Lipofectamine 3000 (Invitrogen, Waltham, MA, USA) for 48 h. RT-qPCR was performed to test the transfection efficiency. The transfection sequences are listed in Supplementary Table S1.

### RT-qPCR

Total RNA was extracted from the collected clinical specimens and RB cells using TRIzol (Invitrogen, USA). The dose and purity of the RNA samples were assessed with an A260/A280 ratio >1.8. Next, 2 µg RNA was reverse transcribed into cDNA using the miRcute miRNA First-Strand cDNA Synthesis Kit (Tiangen, Beijing, China) or BlazeTaq SYBR Green qPCR Mix Kits (GeneCopoeia, Rockwille, MD, USA). The expression of the indicated genes was quantified using the TaqMan real-time PCR system (Thermo Fisher Scientific, USA). The results were normalized to glyceraldehyde-3-phosphate dehydrogenase (GAPDH) or U6 snRNA (U6) according to the 2^−ΔΔCt^ method [18]. The primers used are listed in Table 1.

**Table 1.** The sequences of the primers in this study

Primer	Sequences
**AFAP1-AS1**	Forward: 5′-TCGCTCAATGGAGTGACGGCA-3′
	Reverse: 5′-CGGCTGAGCCGCTGAGAACTT-3′
**GNB1**	Forward: 5′-AGGGGTAAGGGAGCAGAG-3′
	Reverse: 5′-GCAGCAGTAGTGGCTTCTCC-3′
**miR-545-3p**	Forward: 5’-CGACAAGGGTCAGCAAACATT-3’
	Reverse: 5’-GCAGGGTCCGAGGTATTC-3’
**GAPDH**	Forward: 5’-TGTTCGTCATGGGTGTGAAC-3’
	Reverse: 5’-ATGGCATGGACTGTGGTCAT-3’
**U6**	Forward: 5’-CTCGCTTCGGCAGCACA-3’
	Reverse: 5’-AACGCTTCACGAATTTGCGT-3’

### Western blot

Cells were lysed using radioimmunoprecipitation assay lysis buffer and microcentrifuged for 10 min. The supernatant was prepared and subjected to detection of protein concentration using the Bio-Rad protein assay dye reagent (Hercules, CA, USA). Protein samples (30 μL) were separated at a constant voltage of 160 V on 15% sodium dodecyl sulfate-polyacrylamide gel electrophoresis (SDS-PAGE) and electro-transferred onto a polyvinylidene fluoride membrane at a constant voltage of 30 V overnight at 4°C. After blocking by blocking buffer for 1 h at room temperature with agitation, the membrane was incubated with GNB1 (ab137635, 1:5000, Abcam, UK) and GAPDH (ab8245, 1:1000, Abcam) antibodies overnight at 4°C, and then incubated with horseradish peroxidase-conjugated secondary antibody (ab205718, 1:2000, Abcam) at 37°C for 1 h. Finally, protein expression was detected using an ECL chemiluminescent detection system (GE Healthcare, Chicago, IL, USA) and quantified using ImageJ software (National Institute of Health, Bethesda, MD, USA) [19].

### Cell counting kit-8 (CCK-8) assay

Cells (1 × 10^4^ cells/well) were cultured in 96-well plates. After 24 h, 48 h, and 72 h, the CCK-8 reagent was supplemented and co-cultured with the cells for 1 h. The plates were read with a microplate reader at 450 nm (Reagene, Shenzhen, China) [20].

### Transwell migration assay

In this experiment, 8 μm Millicell cell culture inserts (Millipore, Burlington, MA, USA) were used to detect cell migratory capacity. After trypsinization, the cells were suspended in medium and added to the inserts. The inserts were placed in a 24-well plate containing 500 μL of 20% FBS medium in each well. After incubation in a cell culture incubator for 2.5 h, the Transwell inserts were removed. The cells that migrated through the bottom surface of the insert were fixed with methanol and stained with toluidine blue before cell calculation and imaging under a light microscope [19].

### Measurement of caspase-3 activity

A caspase-3 activity assay kit (BioVision, China) was used to measure caspase-3 activity. First, the collected cells were processed in lysis buffer containing a protease inhibitor cocktail (Merck, Darmstadt, Germany) for 30 min. After centrifugation, the supernatant containing the total protein samples was prepared for cell lysates. Protein samples (5 µL) were treated with Ac-DEVD-AMC agent and read using a Polarstar spectrofluorimeter at 460 nm [21].

### Luciferase reporter assay

Luciferase reporter plasmids were obtained from GenePharma (Shanghai, China). The wild-type (WT) fragment of AFAP1-AS1 and 3′-UTR GNB1 containing binding sites of miR-545-3p were fused into pGL3 plasmids, named as AFAP1-AS1-WT and GNB1-WT, respectively. In addition, the corresponding mutant luciferase reporter constructs carrying the corresponding mutant (MUT) sequence of AFAP1-AS1 and 3′-UTR were also generated and termed as AFAP1-AS1-MUT, GNB1-MUT1, GNB1-MUT2, and GNB1-MUT1+ MUT2. For luciferase reporter assays, 1 × 10^4^ Y-79 and WERI-Rb-1 cells were transfected with miR-545-3p mimic or mimic-NC with the indicated luciferase reporter vectors for 48 h. Luciferase activity was recorded using a GloMax-96 Microplate Luminometer (Promega, Madison, WI, USA). The luciferase/renilla signal ratio was calculated and defined as the relative luciferase activity [22].

### RNA pull down

Biotin-miR-545-3p (Bio-miR-545-3p) and its corresponding negative control (Bio-NC) were provided by RiboBio Co. Ltd., (Guangzhou, China). First, bio-miR-545-3p or bio-NC were introduced into 1 × 10^5^ Y-79 and WERI-Rb-1 cells. After 24 h, the cells were lysed and incubated with streptavidin beads. RT-qPCR was performed to detect enrichment in the pull-down Biotin – miRNA–mRNA complexes [23].

### RNA immunoprecipitation (RIP)

To further confirm the target bound by miR-545-3p, RNA immunoprecipitation was performed using an RNA immunoprecipitation kit (Geneseed Biotech, Co. Ltd., Guangzhou, China) as instructed. 1 × 10^5^ Y-79 and WERI-Rb-1 cells at 75% confluence were transfected with miR-545-3p mimic or mimic-NC. At 48 h post-transfection, the cells were lysed in RIP lysis buffer. The supernatant was prepared by centrifugation and incubated with 10 µL anti-Ago2 antibodies and normal IgGs at 4°C for 2 h following another 2 h incubation with 40 µL of protein A/G beads. Successful immunoprecipitation of miR-545-3p-related RNAs was evaluated by RT-qPCR [24].

### Animal xenograft experiment

All experimental animal protocols were approved by the Institutional Animal Care and Use Committee of the Nuclear Industry 416^th^ Hospital, Chengdu 610,051, Sichuan, China. BALB/c nude mice (six-week-old, female, two mice) purchased from Hunan SJA Laboratory Animal (China) were housed under specific pathogen-free conditions. WERI-Rb-1 stably transfected with antagomiR-545-3p or antagomiR-NC (GeneCopoeia, USA) were inoculated into the right-back of nude mice (2 × 10^6^ cells/mouse), with antagomiR-NC as a control. The xenograft volume was examined with digital calipers according to the formula volume = 1/2 (length × width^2^) every 3 days after the xenograft model was established for 12 days. After 33 days, the mice were sacrificed, and xenograft tumors were resected and weighed [25].

### Statistical analysis

Data were presented as mean ± standard deviation of three independent experiments.

Statistical analyses were performed using Prism 9.0. Statistical significance was set at p < 0.05. For group comparisons, Student’s t-test was applied. For multiple groups, statistical differences were determined using analysis of variance (ANOVA). The correction of miR-545-3p with AFAP1-AS1 or GNB1 was evaluated using the Pearson method.

## Results

Bioinformatic analysis was used to identify AFAP1-AS1, miR-545-3p, and GNB1 as the genes to be studied. A series of biological function studies revealed that AFAP1-AS1 and GNB1 play a carcinogenic role in RB, whereas miR-545-3p acts as a tumor suppressor. Furthermore, we found that AFAP1-AS1 in RB targets miR-545-3p and releases GNB1, suggesting that the AFAP1-AS1/miR-545-3p/GNB1 axis may be a potential regulatory network of RB.

### AFAP1-AS1 may regulate RB progression by regulating miR-545-3p and GNB1

**Figure 1.**
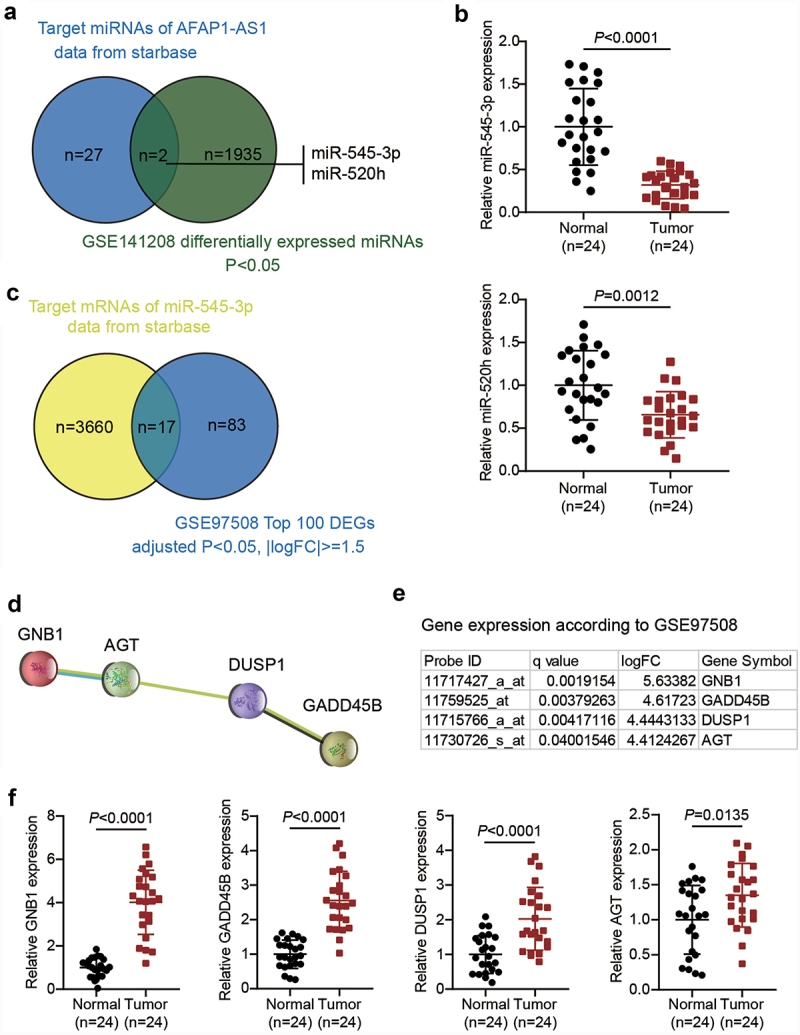
AFAP1-AS1 may regulate RB progression by regulating miR-545-3p and GNB1. (a). The common miRNAs that are both the target miRNAs of AFAP1-AS1 and differentially expressed in RB according to GSE141208 data analysis. (b) RT-qPCR analyzing miR-545-3p, miR-520 h expression in RB tissues (N = 24). Repetition = 3. (c). The common mRNAs that are both the target mRNAs of miR-545-3p and differentially expressed in RB according to GSE97508 data analysis. (d). The protein-protein interaction network analysis of the 17 common mRNAs from figure C. (e). The expression levels of the four genes in the PPI network according to GSE97508. (f) RT-qPCR analyzing GNB1, GADD45B, DUSP1, AGT expression in RB tissues (N = 24). Repetition = 3.

AFAP1-AS1 has been reported to be a cancer driver in RB [12]. However, studies on AFAP1-AS1 in RB are limited. By predicting the target miRNAs of AFAP1-AS1 using the starBase algorithm and analyzing the DEGs of GSE141208, we identified two miRNAs, miR-545-3p and miR-520 h (Figure 1(a)). miR-545-3p has been reported to be a significant tumor suppressor in human cancers other than RB [26,27]. In addition, the qRT-PCR analysis showed that the expression of miR-545-3p and miR-520 h in RB was downregulated by approximately 70% and 40%, respectively. Hence, miR-545-3p was selected as the miRNA of interest (Figure 1(b)). By predicting the targets of miR-545-3p using starBase and analyzing GSE97508 (the top 100 significantly upregulated genes were used), we identified 17 common mRNAs (Figure 1(c)). On uploading the 17 genes to the STRING database, a protein-protein interaction network of four of the 17 genes was obtained, as shown in Figure 1(d). Among the four genes, GNB1 showed the most significant upregulation (logFC = 5.63382) in RB, according to GSE97508 data (Figure 1(e)). Similarly, qRT-PCR results showed that levels of GNB1, growth arrest and DNA damage-inducible beta (GADD45B), dual specificity phosphatase 1 (DUSP1), and angiotensinogen (AGT) were 4.0, 2.5, 2.1, and 1.3 times higher in RB tissues than in normal tissues, respectively. Therefore, we continued to study the regulation of AFAP1-AS1, miR-545-3p, and GNB1 in RB (Figure 1(f)).

### *AFAP1-AS1 drives tumourigenicity of RB* in vitro

**Figure 2.**
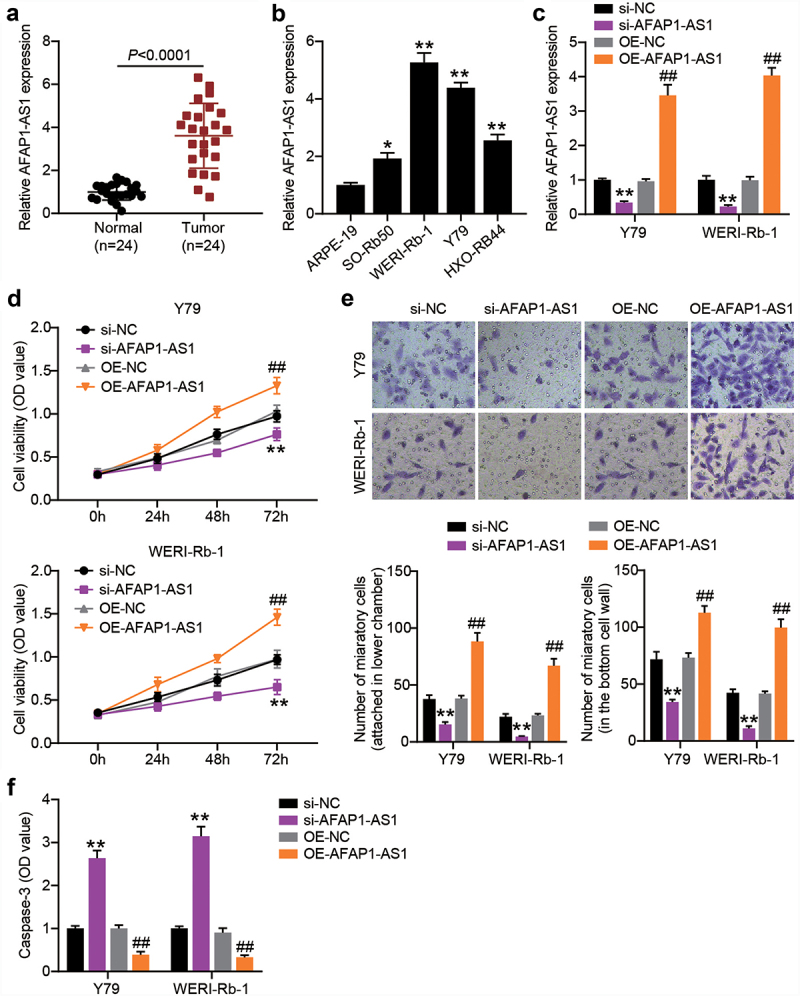
AFAP1-AS1 affects RB cell proliferation, migration and casepase-3 activity. RT-qPCR analyzing AFAP1-AS1 expression in RB tissues (N = 24) (a) and cells (b) **P < 0.001 vs. normal tissues or normal cells. (c) Y79 and WERI-Rb-1 cells were transfected with sh-AFAP1-AS1, OE-AFAP1-AS1 vectors or their corresponding NC (sh-NC and OE-NC) for 48 h. For AFAP1-AS1 overexpression, puromycin-resistant colonies were selected. RT-qPCR analysis was adopted to verify the AFAP1-AS1 silence or enforced expression. (d) CCK8 assays following AFAP1-AS silence and overexpression. (e) Transwell migration assays following AFAP1-AS silence and overexpression. (f) Comparison of casepase-3 activity in RB cells following AFAP1-AS silence and overexpression. **P < 0.001 vs. si-NC^. ##^P < 0.001 vs. OE-NC. N = 3, repetition = 3.

AFAP1-AS1 is a critical tumor promoter gene in RB [12]. To further explore the role of AFAP1-AS1 in RB, we first compared the expression of AFAP1-AS1 in RB tissues and normal tissues. As shown in Figure 2(a), AFAP1-AS1 expression was upregulated in RB tissues. Thereafter, the relationship between AFAP1-AS1 expression and the clinicopathological parameters of patients with RB was analyzed. We found that AFAP1-AS1 expression was not associated with age, gender, differentiation grade, or the largest tumor base. However, high expression levels of AFAP1-AS1 were closely associated with TNM stage and optic nerve invasion (Table 2). The above data suggest that upregulated AFAP1-AS1 expression in RB is correlated with cancer progression. In addition, higher expression of AFAP1-AS1 was also detected in RB cells, especially in Y-79 and WERI-Rb-1 cells (Figure 2(b)). Thereafter, we used gain-of-function and loss-of-function assays in Y-79 and WERI-Rb-1 cells to verify the role of AFAP1-AS1 in RB. After the introduction of pcDNA3.1-AFAP1-AS1 plasmids, AFAP1-AS1 shRNA or their NCs into Y-79 and WERI-Rb-1 cells, RT-qPCR analysis was performed to confirm the overexpression or silencing of AFAP1-AS1 in both the RB cells (Figure 2(c)). As shown in Figure 2(d), the AFAP1-AS1 knockdown resulting from shRNA transfection impaired the proliferation of RB cells, whereas the AFAP1-AS1 overexpression caused by transfection of pcDNA3.1-AFAP1-AS1 plasmids boosted cell proliferation. In addition, shRNA silencing of AFAP1-AS1 reduced the migration of Y-79 and WETRI-Rb-1 cells, whereas the ectopic expression of AFAP1-AS1 resulted in a prominent increase in cell migration (Figure 2(e)). In addition, caspase-3 activity was increased when AFAP1-AS1 was silenced but diminished when AFAP1-AS1 was overexpressed (Figure 2(f)).

**Table 2.** Correlations between AFAP1-AS1, miR-545-3p and clinical characteristics in retinoblastoma patients

Clinicopathological parameters	Number (N = 24)	AFAP1-AS1 expression		P	miR-545-3p expression		P
		High	Low		High	Low	
Age (years)				0.317			1.000
≥ 5	5	1	4		3	2	
< 5	19	11	8		9	10	
Gender				0.414			0.100
Male	13	8	5		9	4	
Female	11	4	7		3	8	
Differentiation				0.193			0.667
Well and moderate	16	6	10		7	9	
Poor	8	6	2		5	3	
TNM stage				0.009			0.009
I+ II	9	1	8		1	8	
III+IV	15	11	4		11	4	
Optic nerve invasion				0.003			0.036
Negative	10	1	9		8	2	
Positive	14	11	3		4	10	
Largest tumor base (mm)				0.371			0.069
≥ 15	7	5	2		1	6	
< 15	17	7	10		11	6	

Fisher’s exact test was used.

### AFAP1-AS1 engages in the modulation of miR-545-3p expression

**Figure 3.**
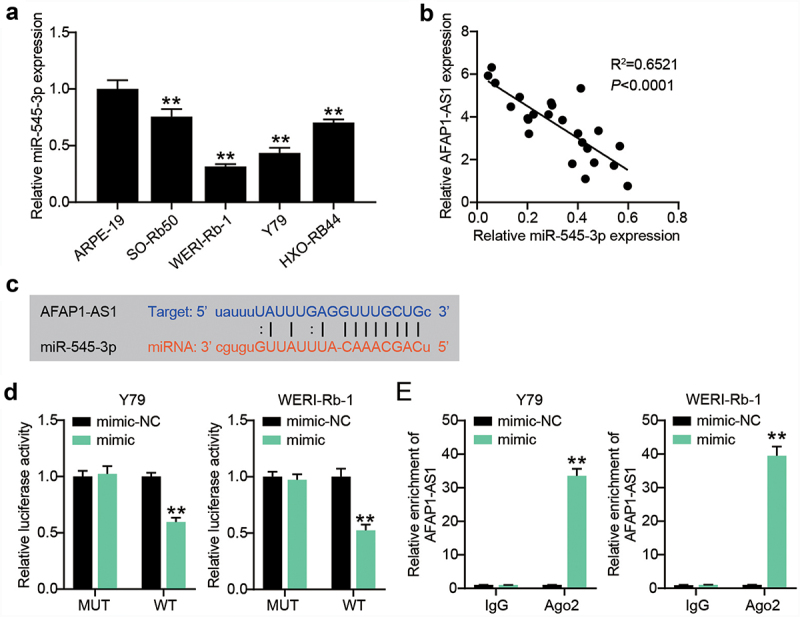
AFAP1-AS1 engages in the modulation of miR-545-3p expression. (a) RT-qPCR analyzing miR-545-3p expression in RB cells. **P < 0.001 vs. ARPE-19 group. (b) Pearson’s correlation analysis of the correlations of AFAP1-AS1 with miR-545-3p in RB tissues. (c) Putative miR-545-3p binding site in the sequence AFAP1-AS1. (d) Luciferase assay. Y79 and WERI-Rb-1 cells were transfected with miR-545-3p or mimic-NC, then transfected with the luciferase constructs of AFAP1-AS1-wt or AFAP1-AS1-mut. The luciferase activity was analyzed. **P < 0.001 vs. mimic-NC group. (e) Ago IP enrichment of AFAP1-AS1 of the miR-545-3p-overexpressing Y79 and WERI-Rb-1 cells. **P < 0.001 vs. mimic-NC group. N = 3, repetition = 3.

It is recognized that lncRNAs play a role as ceRNAs of microRNAs. In our assays, we focused on miR-545-3p, since its interaction with AFAP1-AS1 has been reported in endometrial carcinoma and lung cancer [13,14]. By analyzing the relationship between miR-545-3p expression and the clinicopathological parameters of RB patients, we found that low expression levels of miR-545-3p were closely associated with TNM stage and optic nerve invasion (Table 2). *In vitro*, we also detected a significantly reduced expression of miR-545-3p in RB cells (Figure 3(a)). The lowest miR-545-3p expression was observed in Y-79 and WERI-Rb-1 cells. The expression of miR-545-3p is inversely correlated with AFAP1-AS1 expression in RB tissues (Figure 3(b)). Subsequent starBase analysis further verified that AFAP1-AS1 shared potential binding sites with miR-545-3p (Figure 3(c)). The luciferase assay system demonstrated that miR-545-3p overexpression by mimic transfection impaired the transcriptional activity of AFAP1-AS1; however, it had no effect on the AFAP1-AS1 mutant transcriptional activity (Figure 3(d)). Furthermore, the Y-79 and WERI-Rb-1 cells with miR-545-3p mimics manifested AFAP1-AS1 enrichment compared to the IgG control (Figure 3(e)). These findings suggest that downregulation of miR-545-3p might partially account for AFAP1-AS1 regulation with its ceRNA activity.

### miR-545-3p-downregulation restores the effect of AFAP1-AS1 silence on proliferation and migration in Y-79 and WERI-Rb-1 cells

**Figure 4.**
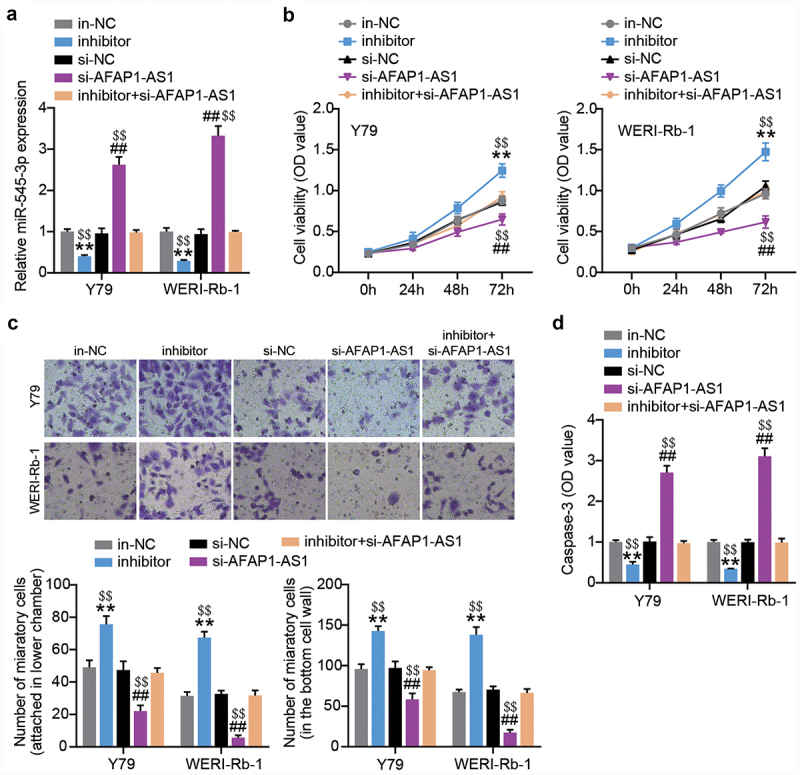
AFAP1-AS1 upregulates miR-545-3p to inhibit proliferation and migration in RB cells. MiR-545-3p inhibitor, inhibitor NC, si-AFAP1-AS1 and si-AFAP1-AS1+ miR-545-3p inhibitor were introduced into Y-79 and WERI-Rb-1 cells. After transfection of 48 h. (a) RT-qPCR analysis of the miR-545-3p expression. (b) CCK8 assays detecting cell proliferation. (c) Transwell migration assay detecting cell migration. (d) Casepase-3 activity was determined. **P < 0.001 vs. in-NC group, ##P < 0.001 vs. si-NC group, $$P < 0.001 vs. inhibitor+si-AFAP1-AS1 group. N = 3, repetition = 3. N = 3, repetition = 3.

AFAP1-AS1 siRNA or miR-545-3p inhibitor was introduced into both RB cells. At 48 h post-transfection, RT-qPCR was performed to confirm successful transfection. As shown in Figure 4(a), the siRNA AFAP1-AS1 successfully increased the expression of miR-545-3p in both RB cells. However, upon co-transfection, the increased miR-545-3p resulting from siRNA AFAP1-AS1 transfection was nearly completely abolished by the miR-545-3p inhibitor. The outcome of the cell proliferation assay showed that the miR-545-3p inhibitor increased the proliferation of RB cells. In addition, the miR-545-3p inhibitor offsets the inhibition of cell proliferation caused by AFAP1-AS1 silencing (Figure 4(b)). We further evaluated cell migration and found that miR-545-3p downregulation increased the number of migrated cells in both RB cells, which was abrogated by the synchronous introduction of AFAP1-AS1 siRNA and miR-545-3p inhibitor (Figure 4(c)). In addition, the anti-miR-545-3p treatment reduced caspase-3 activity, which was restored by the additional transfection of AFAP1-AS1 siRNA (Figure 4(d)). In summary, AFAP1-AS1 reduced miR-545-3p expression to boost the proliferation and migration of RB cells.

### *Down-regulation of miR-545-3p promotes the growth of WERI-Rb-1 cells* in vivo

**Figure 5.**
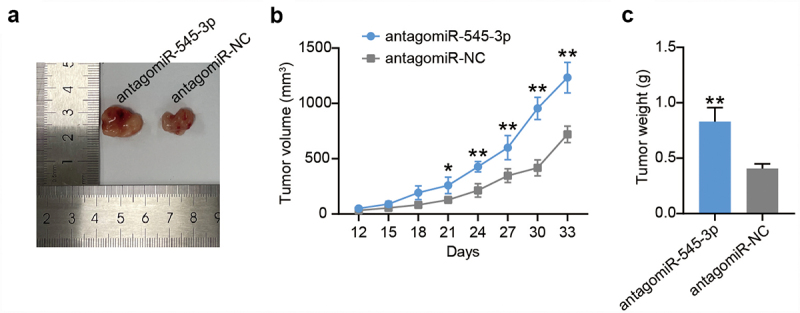
Down-regulation of miR-545-3p promotes the growth of WERI-Rb-1 cells *in vivo*. (a) Subcutaneous transplanted tumors images of mice injected with WERI-Rb-1 cells treated by antagomiR-545-3p (N = 1) or antagomir-NC (N = 1). (b) The tumor volume was examined every 3 days for 33 days. (c) 33 days post-injection, the mice were executed, and tumor weighed was measured. **P < 0.001 vs. antagomiR-NC group. N = 1, repetition = 3.

To verify the role of miR-545-3p *in vivo*, we established a nude mouse model of RB. Nude mice were injected with stable miR-545-3p knockdown WERI-Rb-1 cells, and tumor volume was measured every 3 days for 33 days. At 33 days after injection, the mice were sacrificed, and the tumor was removed. As shown in Figure 5(a), miR-545-3p loss enhanced tumor size compared to the antagomiR-NC group. In addition, compared with the antagomiR-NC group, both tumor volume and tumor weight increased in the antagomiR-545-3p group (Figures 5(b,c)). These results suggest that miR-545-3p loss promotes RB cell growth *in vivo*.

### GNB1 is targeted by miR-545-3p and positively correlated with AFAP1-AS1

**Figure 6.**
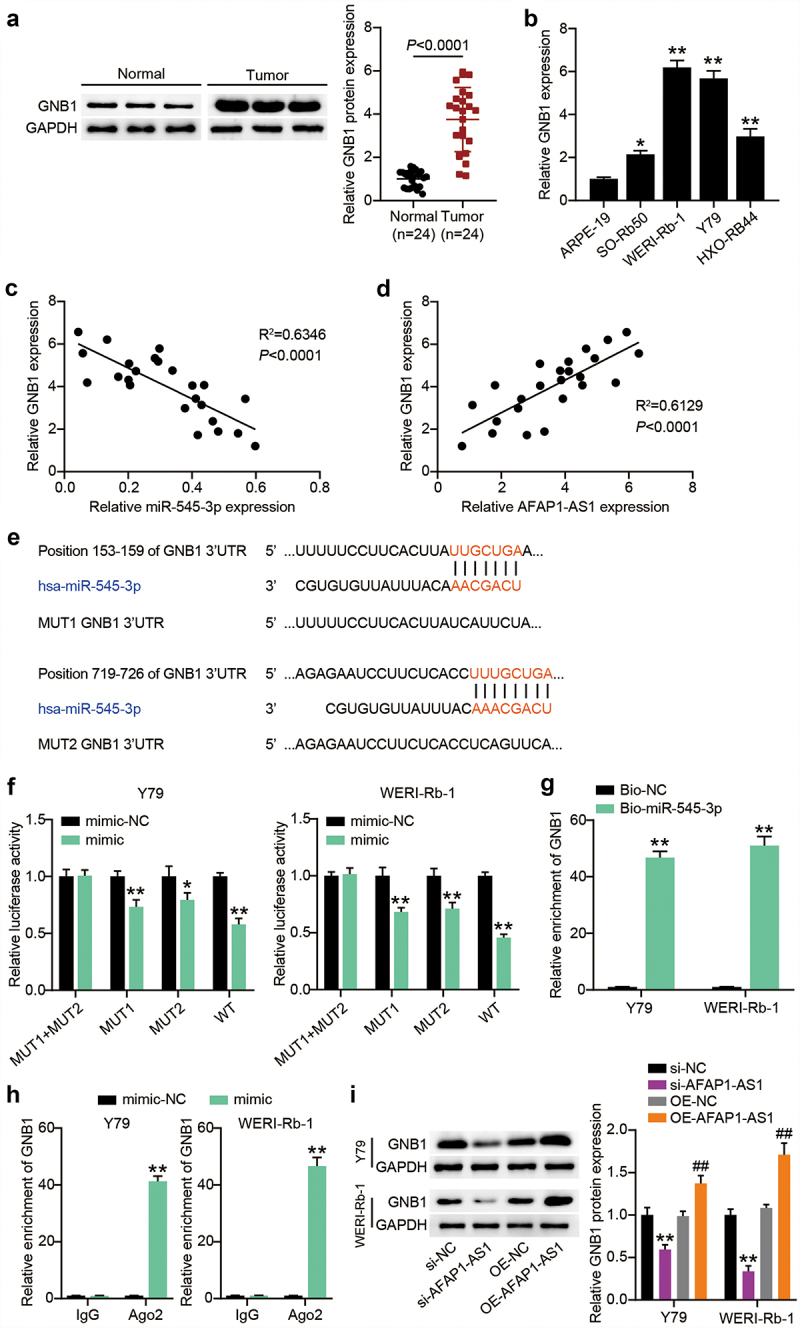
MiR-545-3p directly targets GNB1 1 3ʹUTR. (a) The GNB1 protein levels were detected in GNB1 in RB tissues. (b) The GNB1 mRNA levels were detected in GNB1 in RB cells. *P < 0.05, **P < 0.001 vs. ARPE-19 group. (c) miR-545-3p correlated negatively with GNB1 (Pearson’s analysis, R^2^ = 0.6346). (d) AFAP1-AS1 correlated positively with GNB1 (Pearson’s analysis, R^2^ = 0.6129). (e) Putative binding site of miR-545-3p in the 3ʹUTR region of GNB1. (f) Luciferase activity in Y79 and WERI-Rb-1 cells cotransfected with miR-545-3p mimic or mimic NC and the reporter vectors for 48 h. *P < 0.05, **P < 0.001 vs. mimic-NC group. (g) RNA pull-down analysis was carried out with biotin-labeled miR-545-3p (Bio-miR-545-3p) and its control bio-NC (Bio-NC). Specific primers were used to detect the enrichment of GNB1 by RT-qPCR. **P < 0.001 vs. Bio-NC group. (h) Ago IP enrichment of GNB1 of the miR-545-3p-overexpressing Y79 and WERI-Rb-1 cells. **P < 0.001 vs. mimic-NC group. (i) Y-79 and WERI-Rb-1 cells were transfected with OE-GNB1, si-GNB1 and their corresponding NC for 48 h. Western blots detecting GNB1 protein expression in Y-79 and WERI-Rb-1 cells. **P < 0.001 vs. si-NC group, ##P < 0.001 vs. OE-NC group. N = 3, repetition = 3.

Thereafter, we tested the GNB1 protein expression in RB tissues and found an upregulation of more than 3.7-fold in RB tissues (Figure 6(a)). Downregulation was also detected in RB cells (Figure 6(b)). Notably, a negative correlation between GNB1 and miR-545-3p and a positive correlation between AFAP1-AS1 and miR-545-3p was observed in RB tissues (Figures 6(c,d)). To explore whether GNB1 is a participant of the above predicted ceRNA network, we applied TargetScan to compare the fragment of GNB1 3′-UTR and miR-545-3p and found that GNB1 3′-UTR shared two binding sites with miR-545-3p (Figure 6(e)). We then detected the impact of miR-545-3p on the GNB1 3′-UTR-mediated luciferase activity in Y-79 and WERI-Rb-1 cells with constructs carrying GNB1-WT, GNB1-MUT1, GNB1-MUT2 or GNB1-MUT1+ MUT2. As shown in Figure 6(f), exogenous miR-545-3p overexpression significantly reduced GNB1-WT mediated luciferase activity and partially reduced GNB1-MUT1 or GNB1-MUT2 control luciferase activity while having no effect on GNB1-MUT1+ MUT2 control. To further verify this interaction, RNA pull-down analysis was performed in Y-79 and WERI-Rb-1 cells after bio-miR-545-3p or bio-NC transfection (Figure 6(g)). GNB1 enrichment was observed in the Bio-miR-545-3p group but not in the Bio-NC group. RIP confirmed that Y-79 and WERI-Rb-1 cells with miR-545-3p mimic showed more significant GNB1 enrichment upon treatment with Ago2 antibodies (Figure 6(h)). These data reflect the direct binding of miR-545-3p to GNB1. Furthermore, we examined the effect of AFAP1-AS1 on GNB1 expression. Western blotting showed that GNB1 protein levels in the si-AFAP1-AS1 group decreased by more than 40% compared with those of the si-NC group, and the GNB1 protein level in the OE-AFAP1-AS1 group increased by more than 1.3 times compared to those of the OE-NC group (Figure 6(i)). Thus, GNB1 is targeted by miR-545-3p and is positively regulated by AFAP1-AS1.

### GNB1 silence reduces miR-545-3p inhibitor-induced cell proliferation and migration

**Figure 7.**
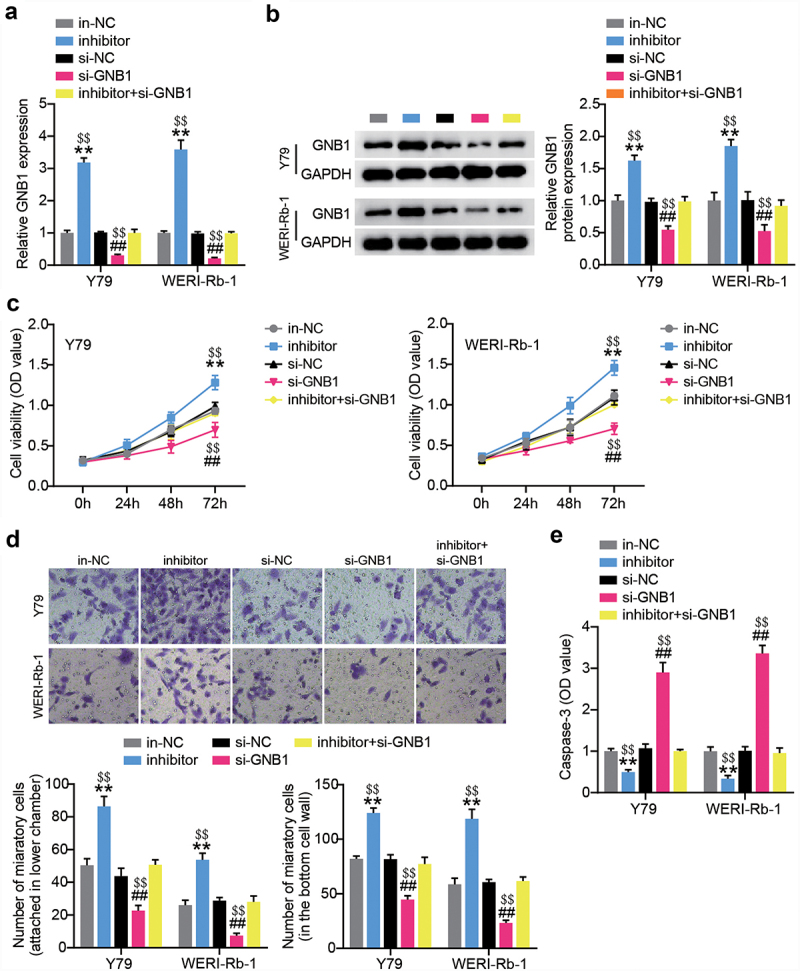
GNB1 silence reduces miR-545-3p inhibitor-induced cell proliferation and migration. Y-79 and WERI-Rb-1 cells were transfected with miR-545-3p inhibitor, si-GNB1 and their corresponding NC for 48 h. RT-qPCR (a) and Western blots (b) detecting GNB1 expression in Y-79 and WERI-Rb-1 cells. (c) Proliferation analysis of Y-79 and WERI-Rb-1 cells by CCK8 assays. (d) migration analysis of Y-79 and WERI-Rb-1 cells by transwell migration assay. (e) casepase-3 activity detection in Y-79 and WERI-Rb-1 cells. **P < 0.001 vs. in-NC group, ##P < 0.001 vs. si-NC group, $$P < 0.001 vs. inhibitor+si-GNB1 group. N = 3, repetition = 3.

Since the interaction between GNB1 and miR-545-3p was verified, we further detected the impact of the loss of GNB1 on the miR-545-3p inhibitor-induced alteration of phenotypes in Y-79 and WERI-Rb-1 cells. We first transfected RB cells with si-GNB1 or miR-545-3p inhibitor. RT-qPCR results demonstrated that si-GNB1 could abolish the increased expression of GNB1 due to endogenous silencing of miR-545-3p by transfecting the miR-545-3p inhibitor (Figure 7(a)). The same expression trend of GNB1 protein was also evidenced by Western blotting (Figure 7(b)). GNB1 silencing reduced cell proliferation and migration. The loss of GNB1 also nearly nullified the miR-545-3p inhibitor-boosted proliferation and migration in Y-79 and WERI-Rb-1 cells (Figure 7(c,d)). In addition, GNB1 silencing of RB cells manifested high caspase-3 activity, whereas simultaneous transfection with si-GNB1 and miR-545-3p inhibitor caused almost no change in caspase-3 activity (Figure 7(e)). All findings suggest that miR-545-3p inhibits proliferation and migration through downregulation of GNB1, accompanied by increased caspase-3 activity.

## Discussion

In this study, AFAP1-AS1 was described as an abundantly expressed lncRNA in RB tissues and cells. Thereafter, loss- and gain-of-function assays demonstrated that ectopically expressed AFAP1-AS1 drives the proliferation and migration of RB cells in addition to inducing caspase-3 activity, whereas the silencing of AFAP1-AS1 by siRNA yielded the opposite results. In addition, AFAP1-AS1 silencing reduced miR-545-3p sponging, thereby reducing GNB1 expression, resulting in curbed RB cell proliferation and migration and increased caspase-3 activity. Hence, the oncogenic role of AFAP1-AS1 was partially regulated by the miR-545-3p/GNB1 axis through its ceRNA activity.

lncRNAs are prominent players in RB tumorigenesis and progression. Xv et al. [28] reported that the expression of XIST was significantly increased in RB cells and that it promoted the proliferation, migration, and invasion of cancer cells. Ni et al. [19] revealed the inhibitory effect of ANT1 on RB progression. In addition, the function of AFAP1-AS1 in RB has been previously reported. Amplified AFAP1-AS1 expression is associated with the clinicopathological features of aggressive RB, and loss of AFAP1-AS1 function inhibits RB growth [12]. In line with previous investigations, we also found abundant expression of AFAP1-AS1 in RB tissues. The promoting effect of AFAP1-AS1 on RB cell proliferation and migration was further corroborated by the loss-of-function and gain-of-function assays. Our findings indicated an oncogenic role of AFAP1-AS1 in RB.

To further explore the regulatory mechanism by which AFAP1-AS1 exerts its oncogenic function, the starBase prediction was used to predict that miR-545-3p has perfect miRNA complementarity with AFAP1-AS1. miR-545-3p plays a tumor-suppressive or oncogenic role in various cancers. For example, in hepatocellular carcinoma, miR-545-3p overexpression facilitates cancer cell viability *in vivo* and *in vitro* [29]. The tumor-suppressive activities of miR-545-3p have been identified in epithelial ovarian cancer and lung cancer [26,27]. However, little has been reported on the same for RB. Our data demonstrated that anti-miR-545-3p treatment resulted in weakened proliferative and migratory capacities and empowered caspase-3 activity, which highlights its suppressive role in RB. In addition, miR-545-3p was poorly expressed in RB tissues and cells and showed a negative correlation with AFAP1-AS1 expression. Luciferase reporter assays coupled with RIP assays in RB cells confirmed the direct interaction of AFAP1-AS1 and miR-545-3p. Their correlation was further reinforced by the negative correlation between miR-545-3p and AFAP1-AS1 in RB tissues. Previous studies have emphasized the oncogenic function of AFAP1-AS1 as an upstream regulator of miR-545-3p in the tumorigenesis of endometrial carcinoma and lung cancer [13,14]. We also investigated the regulation of RB cell phenotypes. The results showed that anti-miR-545-3p treatment can reverse the reduced proliferation and migration caused by AFAP1-AS1 silencing and facilitate RB cell growth *in vivo*. Our findings indicate that AFAP1-AS1 exerts its oncogenic role in RB by sequestering miR-545-3p.

Previous studies have shown that the miR-545-3p-mediated ceRNA activities of AFAP1-AS1 facilitates target depression, such as vascular endothelial growth factor (VEGF) A or hepatoma-derived growth factor [13,14]. This study found that GNB1 might be a target of miR-545-3p in RB. Interestingly, GNB1 is depicted as a tumor suppressor in clear cell renal cell carcinoma, although it is an oncogenic protein in lung cancer and cervical squamous cell carcinoma. GNB1 is a target gene of miR-326 that promotes malignant phenotypes in lung and cervical squamous cell carcinoma and is positively regulated by circ_POLA2 [16,17]. Furthermore, analysis of renal clear cell carcinoma showed that GNB1 was correlated with WASP family member 2 (WASF2), Neuropilin-1 (NRP1) and huntingtin interacting protein 1 (HIP1) of the vascular endothelial growth factor (VEGF) signaling pathway [15]. However, the role and regulatory mechanism of GNB1 in RB remain unknown. Our data showed that GNB1 was amplified in RB tissues and cells. Moreover, GNB1 silencing diminished the malignant behavior of RB cells. The direct relationship between miR-545-3p and GNB1 was confirmed by luciferase reporter and RNA pull-down assays. The miR-545-3p inhibitor-mediated enhancement of RB cell proliferation and migration was also nullified by the addition of si-GNB1. Therefore, GNB1 might participate in the AFAP1-AS1 and miR-545-3p regulatory networks.

It is worth noting that the underlying mechanism of AFAP1-AS1 in RB might involve a more complex network. Further investigations are required to explore this issue. In addition, the tumorigenesis of AFAP1-AS1 requires further verification through *in vivo* experiments. We will focus on developing downstream signaling pathways of the AFAP1-AS1/miR-545-3p/GNB1 regulatory network in the future.

## Conclusion

In summary, we verified the lncRNA-miRNA-mRNA regulatory network for AFAP1-AS1 in RB. AFAP1-AS1 is abundantly expressed in RB tissues. AFAP1-AS1 enhanced proliferation and migration and decreased caspase-3 activity in RB cells. However, AFAP1-AS1 silencing reduced the malignant behavior of RB cells, which was reversed upon treatment with the miR-545-3p inhibitor. In addition, through the interplay with miR-545-3p, AFAP1-AS1 enhanced the expression of GNB1 and facilitated RB cell proliferation and migration. These findings suggest that AFAP1-AS1 is a putative oncogenic lncRNA during RB malignancy, partially via its ceRNA activities that regulate the miR-545-3p/GNB1 axis.

## Supplementary Material

Supplemental Material
