# Supplementary material for: AFAP1 antisense RNA 1 promotes retinoblastoma progression by sponging microRNA miR-545-3p that targets G protein subunit beta 1
Source: Bioengineered. 2022 Feb 22;13(3):5638–52. doi: 10.1080/21655979.2022.2033464 (PMC8974164; doi:10.1080/21655979.2022.2033464)
Supplement: Supplemental Material [file KBIE_A_2033464_SM0001.docx]

Supplementary Table1. Transfection sequences.

| Characteristic | Sequences (5’-3’) |
| --- | --- |
| si-AFAP1-AS1 | AACACCAATCCCAAGAGGTGA |
| si-NC | TTCTCCGAACGTGTCACGT |
| si-GNB1 | CATTATCTGTGGTATCACA |
| miR-545-3p inhibitor | GCACACAAUAAAUGUUUGCUGA |
| inhibitor-NC | AACCUUUAGGGUUCUAGGGAGG |
